# A multimedia tool for infection prevention and control practices in the intensive care unit: a participatory interventional before–after study

**DOI:** 10.1016/j.infpip.2024.100423

**Published:** 2024-12-05

**Authors:** Sunil Kumar Bijarania, Rupinder Kaur, Manisha Biswal, Sangeeta Maheshwar, Rajarajan Ganesan, Goverdhan D. Puri, Sushant Konar, Shyam Thingnam

**Affiliations:** a Department of Anesthesia and Intensive Care, PGIMER, Chandigarh, India; b Department of Medical Microbiology, PGIMER, Chandigarh, India; c Data Entry, PGIMER, Chandigarh, India; d Cardio Thoracic and Vascular Surgery, PGIMER, Chandigarh, India

**Keywords:** Ventilator-associated pneumonia, Central-line-associated bloodstream infection, Infection prevention and control, Cardiac surgical intensive care unit, Healthcare-associated infections, Multimedia tool

## Abstract

**Background:**

Infection prevention and control (IPC) practices by critical care nurses are crucial in preventing ventilator-associated pneumonia (VAP) and central-line-associated bloodstream infection (CLABSI).

**Aim:**

To implement an integrative approach to developing a set of IPC practices and disseminating information on the IPC practices through an educational multimedia tool to improve compliance with the practices.

**Methods:**

This participatory interventional before–after study was conducted in a single tertiary care centre's cardiac surgical intensive care unit (ICU) from May 2022 to March 2023. Thirty-seven nursing IPC practices related to VAP and eight for CLABSI were finalized through a three-step process: systematized review, focused group discussions (five rounds), and Delphi rounds (three rounds). The IPC practices were disseminated through a multimedia tool, displayed continuously in the ICU. Nurses' compliance with the IPC practices observed directly was compared before and after implementing the multimedia tool.

**Results:**

A total of 6043 observations for practices related to VAP and 1957 observations for those of CLABSI were performed. There was an increase in compliance post implementation for 11 IPC practices related to VAP and two IPC for those of CLABSI. There was an increase in compliance with practices relevant to chlorhexidine baths, oral care, cuff pressure maintenance, hypertonic saline nebulization, endotracheal suctioning, scrubbing the hub for central line access, and assessment of the central line for removal.

**Conclusion:**

Through a participatory approach, we developed a set of IPC nursing practices for VAP and CLABSI. Implementing a multimedia tool, which encompasses the newly implemented IPC practices, improved compliance with many practices.

## Introduction

Postoperative cardiac surgical patients are prone to acquiring healthcare-associated infections (HAIs) during their intensive care unit (ICU) stay. The incidence of HAIs is 5–20% following cardiac surgery, and the mortality rate increases as high as five times after acquiring HAIs [1]. The most common HAIs in postoperative cardiac surgical patients are ventilator-associated pneumonia (VAP; 50–60%) and central-line-associated bloodstream infection (CLABSI; ∼25%) [1]. Prolonged ICU stay, mechanical ventilation, and handling of the central line by multiple healthcare personnel predispose the patients to these infections. The critical care nurses' infection prevention and control (IPC) practices can be crucial in protecting patients from VAP and CLABSI [2].

Implementing IPC practices has been demonstrated to reduce the incidence of HAIs in the ICU [3,4]. However, the ICU is a complex environment with multiple barriers to IPC practice compliance. Some barriers to the successful implementation of IPC practices include the absence of local guidelines, inadequate knowledge, high workload, other urgent patient needs, inadequate training, difficulty in integrating IPC practices in the workflow, and the unavailability of resources [[5], [6], [7]]. These barriers must be overcome to reduce HAIs successfully.

Participatory research is a methodology in which all the stakeholders' perspectives are considered in the design and conduct of a study [8]. Regarding IPC practices in the ICU, participatory research is crucial to develop a viable set of IPC practices that is comprehensive, relevant, acceptable to all stakeholders, and up-to-date [2]. Once the set of interventions is developed, continuous and effective dissemination of the interventions is needed, which can be achieved through a multimedia tool [9].

Training in IPC practices can be done through various methods, including face-to-face lectures, workshops, audits, problem-based learning discussions, quizzes, multimedia tools, educational games, simulation, virtual reality, etc. [[10], [11], [12]]. Multimedia tools have certain advantages over other tools in short-staffed, low-resource settings. Face-to-face lectures and workshops offer consistent information delivery and promote interaction among the participants. However, scheduling in-person classes and ensuring attendance are challenging in high-workload environments. On the other hand, online and multimedia tools are readily available for access at the nurses' convenience. In a previous study, which implemented a computer-assisted learning package for IPC practices, the usage was higher among the night-duty and weekend staff, indicating the utility of continuous availability of online tools [13]. Assessments, audits, and feedback comprise an essential aspect of ensuring compliance with IPC practices. They are complementary to educational methods after reducing existing knowledge gaps [6]. Education tools on IPC involving self-study modules and pre- and post-questions are also beneficial in improving patient outcomes [14]. Simulations and virtual reality are attractive teaching modalities capable of improving the knowledge of nurses in infection control practices by offering real clinical care situations [10,11]. Simulation-based training in central line insertion has been shown to reduce the costs associated with CLABSI in a previous study [15].

However, virtual reality tools are resource-intensive and not available widely. On the other hand, multimedia tools can still provide a representation of a clinical scenario at a lower cost, without offering a first-hand experience of the intervention.

We hypothesized that developing a set of IPC nursing interventions for VAP and CLABSI through a participatory approach and disseminating information about the interventions through a multimedia tool would increase compliance with the interventions. The primary objective was to compare the nurses' compliance with IPC practices before and after implementing the multimedia tool. The secondary objectives were participatory approach-based preparation of the multimedia tool through a systematized review of literature, focus group discussions, and Delphi rounds, and the comparison of endotracheal aspirate culture and blood culture positivity rates before and after implementation of the multimedia tool.

## Methods

This participatory interventional before–after study was conducted in the cardiac surgical ICU of a single tertiary care centre between May 2022 and March 2023. The cardiac surgical ICU in our institute is a 13-bed unit with seven beds for adult patients and six beds for paediatric patients.

### Preparation of the multimedia tool

The contents of the multimedia tool were selected through three sequential steps: a systematized review, focus group discussions, and Delphi rounds.

**Figure 1:**
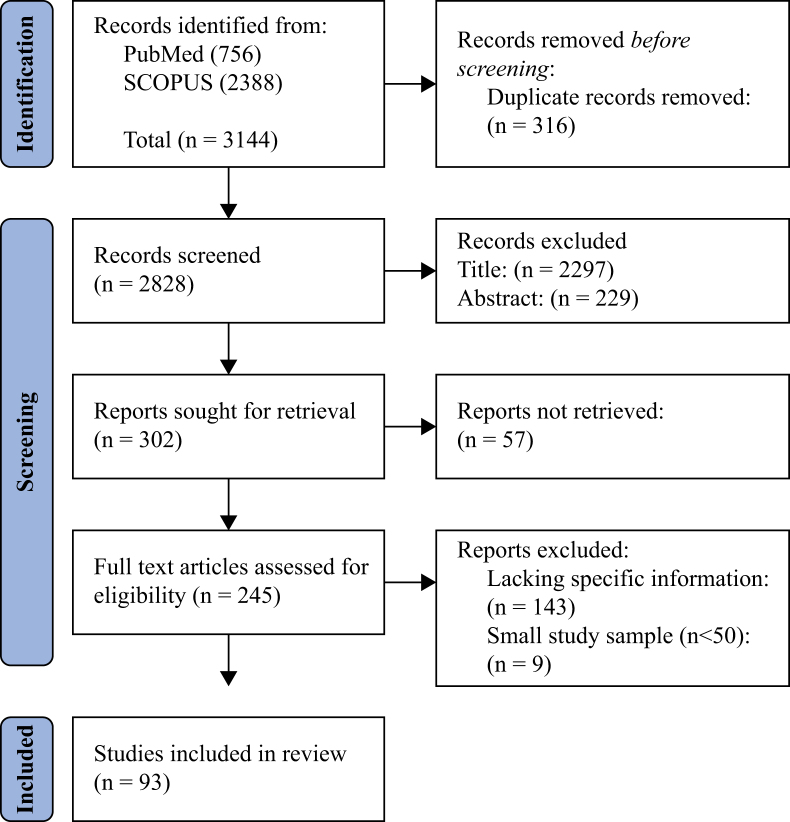
Flow chart of systematized review for ventilator-associated pneumonia.

**Figure 2:**
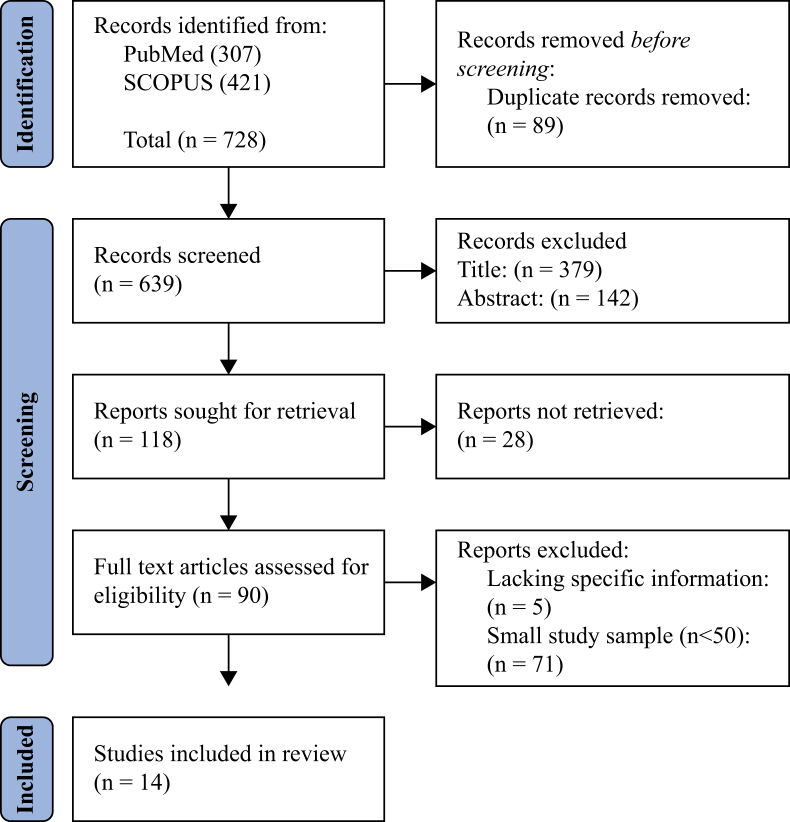
Flow chart of systematized review for central-line-associated bloodstream infection.

Authors S.K.B. and R.G. conducted a systematized review of literature using SCOPUS and PubMed databases to identify all possible IPC practices for nurses. We included studies on VAP and CLABSI in the ICU. Observational studies, clinical trials, and review articles published from January 2007 to June 2022 were included (Figure 1, Figure 2).

A total of 3144 articles were identified for VAP and 728 articles for CLABSI, of which 93 were selected for VAP, and 14 were selected for CLABSI after excluding articles based on title, duplication, and abstract. The systematized review identified 38 IPC practices for VAP and eight IPC practices for CLABSI.

Subsequently, five focus group discussions were conducted in June 2022. The participants included the nursing staff working in the cardiac surgical ICU. A total of 20 nurses were enrolled, with four or five nurses involved per focus group discussion. Each focus group discussion took around 30 min to complete. A semi-structured format was used to focus on the nurses' knowledge of IPC practices, their experience with the current practices and the impediments to implementing the IPCs. The focus group discussions were recorded, and the audio recordings were transcribed, thematically analysed and interpreted. The important findings of focus group discussions were: (1) the unavailability of local VAP and CLABSI prevention intervention bundles or any ready-to-reference material, in case of doubt; (2) limited knowledge about the colonization and transmission of multidrug-resistant pathogens; (3) lack of continuous nursing education; (4) non-compliance with IPC practices due to lack of knowledge and on-the-job training, staffing shortages and heavy workload; (5) the need for administrative reframing of the nurses' role to deploy highly skilled and educated staff effectively, need for ongoing education and incentive programmes; and (6) addition of two new interventions to our IPC practices list: the availability of the hand sanitizer near the patient bed and use of suction support mode on ventilator during suctioning.

The Delphi method was used to obtain a consensus regarding the IPC practices to be included in the multimedia tool. The Delphi panel included 16 members selected purposefully from the fields of critical care nursing (*n* = 4), infection control nursing (*n* = 1), microbiology (*n* = 1), intensive care (*n* = 2), anaesthesia (*n* = 1), and cardiac surgery (*n* = 7) from the study institute and two neighbouring institutions. Delphi rounds were conducted through the SurveyMonkey app between September 2022 and November 2022. The Delphi panel's suggestions were collected using a five-point Likert scale (strongly disagree, disagree, neither agree nor disagree, agree, strongly agree), and one option was provided for additional comment. Separate Delphi rounds were conducted for IPC practices related to VAP and CLABSI. For IPC practices related to VAP, in the first Delphi round, all 16 panelists were requested to assess the content and to suggest their opinions, and 15 panelists replied. One panelist suggested combining three practices into one: ‘Hand hygiene is important in VAP prevention’, ‘Hand hygiene should be performed using an alcohol-based hand rub (if not visibly soiled)’ and ‘Hand hygiene should be performed using soap and water (if visibly soiled)’. All 15 panelists were mailed again for the second round. At this stage, the researcher received replies from 13 panelists. One panelist suggested combining practices, ‘Oral care should be provided with one toothbrush, disposable suction catheter, a disposable syringe, and chlorhexidine/toothpaste’ and ‘An artery forceps and a gauze impregnated with chlorhexidine should be used to cleanse the teeth, tongue, and mucosal surfaces’. One panelist quit in the third round, and the remaining 12 panelists achieved a consensus on the IPC practices related to VAP. All 16 panelists were requested to participate in the Delphi round for IPC practices related to CLABSI. A reply was obtained from 14 panelists with no additions or modifications. The final consensus was obtained in the second round from the 14 panelists. Supplementary Table S1 shows the final list of 37 IPC practices related to VAP and eight IPC practices related to CLABSI and the results of the final Delphi round, respectively [16,17].

For inculcating the IPC practices, videos of the IPC practices were recorded in mid August 2022 in the cardiac surgical ICU (using a professional camera). The ICU nurses performed the procedure on patients, and patients'/relatives' consent was obtained for the recordings. Voice and text titles were edited in the videos after that.

### Observations and implementation of the multimedia tool

The nurses' compliance with the 37 IPC practices related to VAP and eight IPC practices related to CLABSI were assessed before implementing the multimedia tool in September 2022. One researcher unobtrusively observed and recorded the compliance. The observations were conducted on patient beds which had patients either on mechanical ventilation or having an invasive central line *in situ*. The IPC practices were categorized into three parts and a uniform observation protocol was followed to facilitate observation of all the IPC practices and decrease observer bias (Observation sample sheet – Supplementary Appendix): Part 1 included IPC practices for which data was obtained from the patient notes or nurses' report. Whenever oral care, position changes, cuff pressure maintenance, patient bathing, ventilator assessment, and central line assessment were performed by the nurses, it was documented in their report and this was accessible to the researcher. When an IPC practice was mentioned as ‘performed’ for an eligible patient, it was recorded as compliant and vice versa; Part 2 included IPC practices which were observed by the researcher once a day at the bedside. The researcher inspected the availability of sanitizer, patient position, ventilator equipment, and central line site. When it was maintained as per the list of IPC practices, it was recorded as compliant and vice versa. Observation of IPC practices in Parts 1 and 2 was performed between 07:30 and 08:30 every Monday, Wednesday, and Friday for four weeks; Part 3 included procedural practices related to hand hygiene, oral care, endotracheal suctioning, and drug administration through the central line. Hand hygiene compliance was recorded separately only for before patient contact and before each of the procedures of endotracheal suctioning and central line access. Observation for the IPC practices in Part 3 was performed when the procedure was performed by the bedside nurses for 1 h in the evening between 15:00 and 17:00 from Monday to Saturday for four weeks in order to achieve the maximum number of observations. The observation data was recorded online using Google forms.

**Figure 3:**
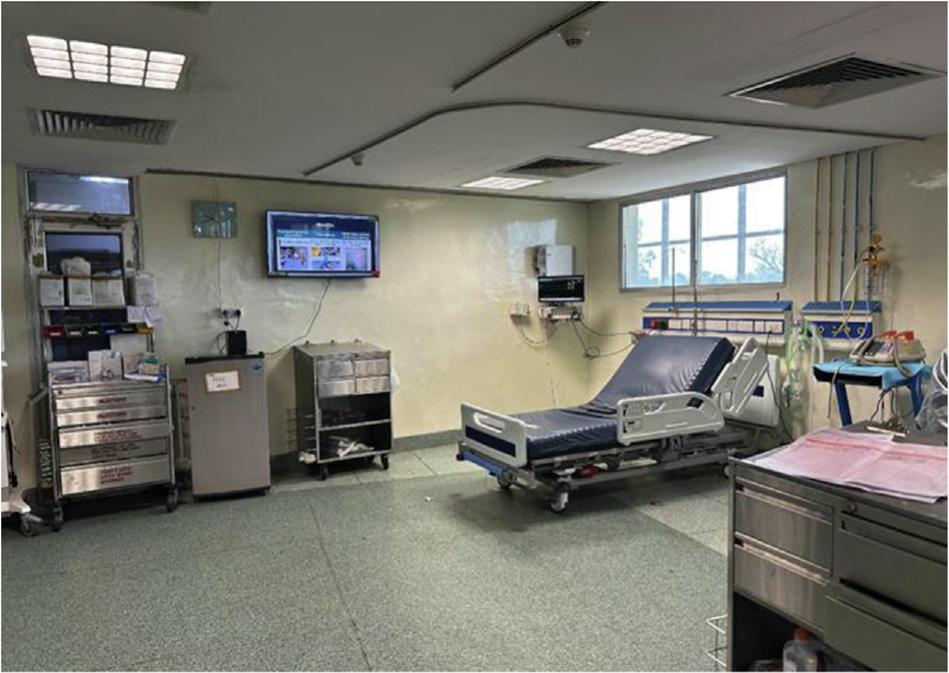
Display of the multimedia tool in the intensive care unit.

The study intervention (multimedia tool implementation) was started in October 2022. The multimedia tool was continuously displayed on a television screen on a wall in the cardiac surgical ICU (Figure 3). All nurses were reminded and motivated to watch the videos during their break and before providing patient care.

Similar to the pre-implementation observation for compliance, the post-implementation compliance was recorded in January 2023. In addition to the compliance data, the data on patient days, mechanical ventilation days, central line days, endotracheal aspirate cultures sent, blood culture samples sent, and positive culture results (after excluding commensals) were recorded. The endotracheal aspirate culture and blood culture were obtained when directed by the treating physician. The endotracheal aspirate was obtained using a suction catheter introduced through the endotracheal tube in a sterile manner and processed immediately. Paired blood cultures were obtained in the patients with a central line after scrubbing the hub or cleaning the venepuncture site with 70% alcohol and leaving to air-dry. Minimum blood volumes of 1, 10, and 20 mL were collected and inoculated in Bactec™ bottles for infants, older children and adults, respectively.

### Statistical analysis

Compliance with the IPC practices before the implementation of the multimedia tool (pre-implementation phase: September 2022) and after the implementation of the multimedia tool (post-implementation phase: January 2023) was compared. The number of observations and the percentage of compliance are reported. Fisher's exact test was used to compare the compliance, and *P* < 0.05 was considered significant. The culture positivity rates for endotracheal aspirate and blood culture samples were calculated by dividing the number of positive cultures by the total number of samples sent during that period [18]. VAP rate was calculated by the formula (Number of positive endotracheal aspirate cultures/Ventilator days) ×1000 and the CLABSI rate was calculated by the formula (Number of positive blood cultures/Central line days) ×1000 [19].

## Results

### IPC practices related to VAP

There was an increase in IPC compliance in the post-implementation phase compared to the pre-implementation phase for 12 IPC practices related to VAP (Table I). There was an increase in compliance for all practices related to bathing the patients with chlorhexidine (three practices) and endotracheal tube cuff pressure assessment (two practices). Similarly, there was an increase in compliance with three of the four practices related to oral care. Compliance with nebulizing paediatric patients with 3% hypertonic saline increased post implementation. Among the practices related to endotracheal suctioning, compliance with three practices – gentle physiotherapy before suctioning, using suction support mode and limiting the time for suctioning – increased post implementation, while compliance with two practices – use of personal protective equipment and wrapping and discarding the suction catheter – decreased post implementation. The compliance was similar between the pre-implementation phase and post-implementation phase for all other practices related to endotracheal suctioning and ventilator care.

**Table I:** Pre- and post-implementation observations and compliance for infection prevention and control practices for VAP

Item no.	Infection prevention and control practices for VAP	Pre implementation			Post implementation			*P*-value (*F*-test)
		No. of observations	Compliance (no.)	Compliance (%)	No. of observations	Compliance (no.)	Compliance (%)	
***Hand hygiene***								
1	A sanitizer bottle is available near each patient's bed	90	83	92.2	64	62	96.9	0.306
2	Hand hygiene is performed using an alcohol-based hand rub (if not visibly soiled) or using soap and water (if visibly soiled)	91	66	72.5	64	49	76.6	0.709
***Oral care***								
3	Patients requiring continued intubation >12 h are provided oral care 6-hourly	87	47	54	64	48	75	0.010
4	The endotracheal cuff pressure is assessed, followed by aspiration of oropharyngeal secretions before beginning the oral care procedure	87	47	54	64	48	75	0.010
5	Oral care is provided with one toothbrush and toothpaste OR an artery forceps and a gauze impregnated with chlorhexidine gluconate to cleanse the teeth, tongue, and mucosal surfaces	84	40	47.6	63	50	79.4	0.001
6	Thereafter oro-pharyngeal area is sucked with a suction set	86	68	79.1	63	51	80.9	0.838
**Daily bath with chlorhexidine gluconate**								
7	Patient (if appropriate post gestational age) admitted to the ICU is bathed/sponged with chlorhexidine solution	86	55	63.9	64	55	85.9	0.003
8	Bathing/sponging is performed daily from admission to discharge	85	53	62.3	64	56	87.5	0.001
9	The whole body surface except for the face, surgical wound and drain tube sites is cleaned in sequence	86	51	59.3	64	50	78.1	0.021
***Assessment of ETT cuff pressure***								
10	ETT cuff pressure is maintained >20 and <30 cmH_2_O with no/minimal leak	84	55	65.5	64	55	85.9	0.007
11	ETT cuff pressure is monitored in each shift in addition to following intubation, after manipulation or adjustment of the endotracheal tube, and when clinically indicated for an air leak or a loss of tidal volume	87	41	47.1	64	49	76.6	0.001
***Hypertonic saline nebulization***								
12	Nebulizing pediatric patients with 3% hypertonic saline	86	55	63.9	64	56	87.5	0.001
13	Hypertonic sodium chloride 3% solution is delivered to the ventilator by placing a T-piece connector into the inspiratory arm of the respiratory circuit around 20 cm from the endotracheal tube	85	47	55.3	64	45	70.3	0.088
***Mechanical ventilation and care***								
14	The ventilator-humidification device is routinely inspected	83	50	60.2	93	54	58.1	0.878
15	The proper temperature setting of the heated humidifier system is maintained	82	58	70.7	93	57	61.3	0.205
16	Condensation from the patient circuit is removed (when required)	83	55	66.3	93	59	63.4	0.752
17	The required water level in the heated humidifier is maintained	83	61	73.5	93	64	68.8	0.51
***Patient position in bed***								
18	Patients' position (if not contraindicated) is changed two hourly	84	56	66.7	93	54	58.1	0.278
19	Pillows/positioning blocks are used to provide right/left lateral position	82	50	61	92	62	67.4	0.429
***Endotracheal suctioning***								
20	Before ET suctioning, manual application of a fine oscillatory movement combined with compression to the patient's chest wall is provided	80	43	53.7	93	87	93.5	<0.001
21	Two appropriately skilled staff perform the suction procedure	82	62	75.6	93	64	68.8	0.399
22	Hand hygiene performed before beginning the procedure	77	62	80.5	93	65	69.9	0.155
23	Use of personal protective equipment	77	66	85.7	92	66	71.7	0.039
24	Suction equipment and appropriate-sized suction catheter (2× ETT internal diameter size) are assembled beforehand	77	65	84.4	93	68	73.1	0.093
25	Perform suction of oral secretions as completely as possible, and do not let the suction tube extend into the oropharyngeal space	76	59	77.6	93	73	78.5	1
26	Position the patient in a semi-Fowler's position (30° bed head elevation) if not contraindicated	76	57	75	93	64	68.8	0.396
27	Pre-oxygenate the patient for two minutes with 100% oxygen (if not contraindicated)	76	59	77.6	93	63	67.7	0.17
28	Sedate the patient with bolus (if agitated)	76	49	64.5	93	59	63.4	1
29	Use in-line suctioning in patients on ventilators for >12 h	72	42	58.3	92	61	66.3	0.33
30	Use suction-support mode for suctioning	76	34	44.7	92	68	73.9	0.001
31	Use the clean hand/dirty hand technique to perform suction	75	60	80	93	71	76.3	0.708
32	Insert a suction catheter in the endotracheal tube until obstruction is felt	74	49	66.2	93	62	66.7	1
33	Suction applied only (100 mmHg) during catheter withdrawal and for no longer than 5 s in each attempt	74	47	63.5	93	66	71	0.322
34	The suction catheter is kept sterile when used for repeated suctions during the same suction episode	74	48	64.9	93	67	72	0.4
35	Wrap and discard the suction catheter if contaminated during the procedure or upon completion of the procedure	74	65	87.8	93	68	73.1	0.021
36	Manually ventilate the patient or apply vital capacity breath (if advised) for lung recruitment after suctioning	75	62	82.7	93	73	78.5	0.561
37	Limiting the time for suctioning <15 s	73	56	76.7	93	84	90.3	0.019

VAP, ventilator-associated pneumonia; ICU, intensive care unit; ETT, endotracheal tube.

### IPC practices related to CLABSI

There was an increase in compliance with two IPC practices related to CLABSI in the post-implementation phase: ‘Scrub the hub’ and daily assessment for central line removal (Table II). The compliance was similar between the pre-implementation phase and post-implementation phase for all other practices related to drug administration through central line and central line maintenance.

**Table II:** Pre-implementation and post-implementation observations and compliance of infection prevention and control practices for CLABSI

Item no.	Infection prevention and control practices for CLABSI	Pre implementation			Post implementation			*P*-value (*F*-test)
		Observations (no.)	Compliance (no.)	Compliance (%)	Observations (no.)	Compliance (no.)	Compliance (%)	
1	Hand hygiene and clean gloving before IV injections	104	54	51.9	145	61	42.1	0.156
2	Scrub the hub with a 70% alcohol swab before accessing it	124	34	27.4	136	70	51.5	0.001
3	Sterile stopper connected to ports which are not in use	102	56	54.9	145	61	42.1	0.052
4	Aspirate from lumen before drug administration	98	60	61.2	137	69	50.4	0.112
5	Any difficulty in flushing the line is checked	100	52	52	141	63	44.7	0.295
6	Intactness of the transparent dressings is checked	112	46	41.1	133	67	50.4	0.158
7	Daily assessment for central line removal	110	46	41.8	129	75	58.1	0.013
8	The central line insertion site is assessed once every shift (for signs of infection)	109	47	43.1	132	68	51.5	0.198

CLABSI, central-line-associated bloodstream infection; IV, intravenous.

### Culture positivity, VAP rate, and CLABSI rate

There was a significant reduction in endotracheal culture positive rate in the post-implementation phase compared to the pre-implementation phase (34.04% vs 59.09% respectively, *P* = 0.0212) while the blood culture positivity rate was not significantly different in the post-implementation phase compared to the pre-implementation phase (9.93% vs 15.62% respectively; *P* = 0.135) (Table III). There were two commensals in blood culture in the pre-implementation phase and four commensals in the post-implementation phase. There was no difference in VAP rate and CLABSI rate before and after the implementation of the multimedia tool.

**Table III:** Endotracheal aspirate culture and blood culture positivity, VAP rate and CLABSI rate

Aspirate/culture	Pre implementation (July–September 2022)	Post implementation (January–March 2023)	*P*-value
Total endotracheal aspirate samples collected	44	47	
Positive endotracheal aspirate culture	26	16	
Culture-positivity rate	59.09%	34.04%	0.021
Ventilator-days	563	394	
Blood cultures positive	25	16	
VAP rate (per 1000 ventilator-days)	46.1	40.6	0.682
Total blood cultures collected	160	161	
Central line-days	722	681	
Culture-positivity rate	15.62%	9.93%	0.135
CLABSI rate (per 1000 central line-days)	34.6	23.5	0.215

VAP, ventilator-associated pneumonia; CLABSI, central-line-associated bloodstream infection.

The total numbers of patient-days, ventilator-days and central-line-days were, respectively, 1035, 563 and 722 in the pre-implementation phase and 840, 394, and 681 in the post-implementation phase.

## Discussion

Multimedia tools for IPC nursing practices for VAP and CLABSI were developed and deployed in this participatory interventional trial. Thirty-seven IPC practices were identified related to VAP and eight IPC practices related to CLABSI through a systematized literature review followed by focus group discussions and Delphi rounds. After deploying the multimedia tool, compliance was increased in 11 IPC practices related to VAP and two IPC practices related to CLABSI. There was also a reduction in the culture positivity rate of endotracheal aspirate following the implementation of the multimedia tool.

The Institute of Healthcare Improvement's Ventilator care bundle is the most commonly implemented list of IPC practices for VAP. It comprises head-end bed elevation, sedation vacation, peptic ulcer prophylaxis, and deep venous thrombosis prophylaxis [20]. There are additional interventions beyond this bundle, such as oral care, cuff-pressure control, and ventilator equipment management, which have been shown to reduce the incidence of VAP in various studies [3,4,21]. Similarly, multiple CLABSI bundles exist, focusing on central line insertion and maintenance [22]. The abundance of practices, heterogeneity among the practices, and need for local feasibility and acceptability are already recognized [2]. In this study, we identified 37 IPC practices related to VAP, which included structural and procedural elements.

Multimedia tools developed by intensive care providers are valuable aids for healthcare education [23]. A recent systematic review of technology-based educational tools for nursing learning outcomes has identified improved knowledge, skill, and self-confidence [24]. We proposed that the improvement in compliance with IPC practices in our study can be attributed to at least three reasons: (1) the multimedia tool served as a ready reference material in case of doubts; (2) it served as training material for inexperienced newcomer nurses; (3) the continuous display of the videos in the ICU could have served as a reminder to maintain IPC practices.

### IPC practices related to VAP

Hand hygiene is recognized as the most important practice for preventing any HAI [25]. Ensuring the placement of hand sanitizer near the patient's bed was an important practice identified during the focus group discussions and was the first practice included in our study [26]. The baseline compliance with hand hygeine was already high in this study, at 92.2%. It increased to 96.9% post implementation of the multimedia tool, though this was not statistically significant. The other IPC practices implemented in our study included frequency and procedure for oral care, daily patient baths with chlorhexidine, cuff-pressure control, nebulization and endotracheal suctioning procedure, all of which are vital in the prevention of VAP.

The compliance with oral care before the implementation of multimedia tools in our study was 54%, and it increased to 75% post implementation (*P* = 0.010). Similar values for compliance with oral care were found in another study, which implemented six IPC practices related to VAP through training and feedback [27]. Moreover, persistent reinforcement of this intervention is required in cardiac surgical patients because the more urgent needs of the patients may compromise their oral care [28]. In our study, toothbrush or gauze was used in less than half of the patients who received oral care in the pre-implementation phase, but this increased to around 80% (*P* < 0.001) in the post-implementation phase. Recent studies highlight the importance of using a toothbrush or gauze in reducing the incidence of VAP [29].

Daily baths with chlorhexidine complement barrier methods in reducing the incidence of VAP and CLABSI [30]. Similar to studies using bathing logs to improve compliance with daily baths, we observed an increase in compliance from 64% to 86% (*P* = 0.003), using our multimedia tool [31].

Maintaining an endotracheal cuff pressure >20 mmHg is a long-recognized IPC practice related to VAP that prevents the occurrence of micro-aspirations [32]. In our study, the compliance with this intervention was 65.5% pre implementation and 85.9% post implementation (*P* = 0.007), similar to values seen in other studies [28,33].

There was an increase in compliance post implementation for nebulization with hypertonic saline in paediatric patients (87.5% from 64%, *P* = 0.001). Nebulization with hypertonic saline reduces the incidence of VAP in paediatric patients by preserving lung clearance [34].

Appropriate endotracheal suctioning is vital to clear lung secretions in a ventilated patient and, at the same time, maintain patient safety [35]. In our study, compliance with ‘use of suction support mode’ and ‘suction duration of <15 s’ increased post implementation (73.9% from 44.7%, *P* < 0.001; and 90.3% from 76.7%, *P* = 0.019), respectively. The suction support mode avoids environmental contamination by discontinuing the cycling of the ventilator during the suctioning. It also ensures continued ventilation after suctioning [36]. Similarly, limiting the suction duration avoids patient desaturation and mucosal irritation.

For two of the endotracheal suction interventions – ‘use of personal protective equipment during suctioning’ and ‘wrapping to discard the suction’ – compliance decreased post implementation compared to pre implementation (71.7% from 85.7%, *P* = 0.039; and 73.1% from 87.8%, *P* = 0.021, respectively). The cause of this decrease is not clear. For other practices related to endotracheal suctioning, the compliance was similar to what has been reported in a previous systematic review [37].

The compliance with IPC practices related to managing the humidification system and routine position was inconsistent the baseline compliance was low; and the compliance did not improve with the multimedia tool. The following are a few possible reasons for the absence of an improvement in compliance. Artificial humidification of the patient's airways is necessary to prevent VAP [38]. Heated humidifiers need to be periodically inspected for correct temperature and adequate water level. At our hospital, the maintenance of heated humidifiers is a collaborative goal between nurses and technicians. We only provided education on the heated humidifier without formally assessing knowledge or implementing a communication protocol which is necessary in a teamwork situation [39]. Similarly, timely emptying of condensate from the tubing into the watertrap also requires collaborative teamwork.

In mechanically ventilated patients, placing the vibrating mesh nebulizer in the inspiratory limb 20 cm from the endotracheal tube has been recommended since it provides maximum aerosol delivery [40]. The optimal position depends on the type of nebulizer, and bedside training may be needed to increase the nurses' familiarity with this practice.

Patient position changes in the form of side-tilts are necessary for pressure ulcer prevention as well as effective drainage of respiratory secretions [41]. In a previous survey among Australian ICUs, compliance to position change was <50% owing to educational and environmental barriers [42]. Haemodynamic stability is a prerequisite for performing position changes. In the cardiac surgical ICU where ensuring continued hemodynamic stability is a challenge, the patient factor could play a major role in affecting compliance.

Among the endotracheal suctioning interventions, we included all steps, including steps not directly related to IPC to ensure a swift procedure without compromising sterility and patient safety. For example, administration of bolus sedation and use of suction support mode were included since failure to perform this timely can result in interruption of the suction procedure to troubleshoot, thereby compromising sterility.

The staff shortage in our ICU limited compliance with the need for the presence of two skilled staff for suctioning. Different teaching tools have been attempted to improve compliance with endotracheal suction interventions. In a previous study, teaching endotracheal suctioning procedures in small groups showed improvement in the knowledge and practice of nurses [43]. On the other hand, a single dose of simulation education failed to show an improvement [44]. Therefore, multimedia tools alone may not be effective in improving all the endotracheal suction practices.

A recent scoping review has identified nurses' barriers to compliance with VAP guidelines [45]. The barriers included work environment, nurse-related and situation-related barriers. Educational support is only one such barrier that can be addressed by the use of our multimedia tool. The other barriers – such as those related to lack of equipment, lack of staff and time and concerns related to the specific adverse events from an intervention on a particular patient – are not addressed by the multimedia tool. Nevertheless, providing education may be crucial in situations where the nurse may otherwise have a positive attitude towards the intervention but lacks knowledge of it. For example, a recent study identified that nurses have moderate knowledge, but also show a higher-than-average level of intervention compared to other ICU interventions for the provision of oral care in intubated patients [46].

Similarly, compliance with hand hygiene before patient contact was around 70% and did not improve post implementation. This is similar to the average compliance with hand hygiene worldwide, which is around 70% [47]. In our study, the compliance with hand hygiene before suctioning and central line access was around 60%. A recent study has identified the use of gloves as a barrier to performing hand hygiene, and that staff often prefer a direct-gloving technique whenever gloves are used [48]. Nevertheless, guidelines still recommend hand hygiene before gloving. Improving compliance with hand hygiene often requires a multimodal approach and frequent feedback [25].

### IPC practices related to CLABSI

The overall compliance with IPC practices related to CLABSI was poor, at around 50%, similar to what has been reported in previous studies [49,50]. However, there was an increase in compliance with the ‘scrub the hub’ practice post implementation (51.5% from 27.4%, *P* < 0.001), thereby validating the utility of our multimedia tool for this intervention.

Unlike IPC practices related to VAP, the IPC practices related to CLABSI can have a higher risk of desensitization due to two reasons. First, the number of times the central line is accessed is more than the number of times ventilator care is provided. Second, VAP is more easily identifiable by the nurses through the presence of secretions than CLABSI, which may not be readily apparent [51].

At our hospitals, three-way stopcocks are used with central venous access. Needle-free connectors are preferred over three-way stopcocks to reduce CLABSI [52]. However, nurses are often not familiar with the correct use of needle-free connectors [53]. When a three-way stopcock is used, the ports of the stopcock can be disinfected with alcohol before use and a sterile cap/stopper is replaced after drug administration [54]. Once the cap/stopper is removed from a port for drug administration, the sterility needs to be maintained until it is replaced on the port or a new sterile stopper needs to be placed. Low compliance was observed with this intervention, probably related to the difficulty in integrating this action into the workflow and the lack of easy availability of additional stoppers. We consider that this can be addressed by using needle-free connectors and providing additional stoppers.

Blocked central venous catheters are predisposed to develop infection [55]. When a central line is *in situ*, failure to aspirate from a lumen but ability to flush indicates a partial occlusion, and failure to also flush indicates a complete occlusion [56]. Nurses play a crucial role in notifying the physician of any occlusion in the lumen by checking for aspiration before drug administration and in preventing any occlusion by flushing the lumen after use. Lack of standard procedure for flushing, individual beliefs about the efficacy of the intervention and the time required to prepare a flushing solution may be some of the barriers that contributed to the absence of improvement in compliance with this intervention [57]. Similarly, the assessment of the central venous line insertion site and ensuring intactness of dressing are also crucial roles played by the nurses. This may not be followed when there is a staff shortage and when there is no defined protocol for documentation [58]. Therefore, the ideal method to ensure compliance with all these interventions could be a stepwise implementation of the interventions in a Plan–Do–Study–Act project where all the barriers are addressed through leadership, cross-department coordination and innovation, apart from education using multiple tools, including simulation [57,59].

The primary strength of our study is its participatory nature, in which we developed the set of practices with the active involvement of the participating nurses whose compliance was measured. This ensured the integration of the nurses' perspectives with the researchers' objectives to produce a viable set of practices [8]. The initial set of practices was developed using a systematized review. In the systematized review, we performed a comprehensive search for all relevant VAP and CLABSI IPC interventions without qualitative assessment [60]. Following this, the focus group discussions elicited the nurses' perspectives. This was vital in ensuring the practicability of the IPC practices and in understanding possible impediments to compliance [61]. Finally, the Delphi rounds helped achieve a consensus among all the stakeholders regarding the IPC practices to be implemented [62]. Another strength of our study is the direct observation for compliance assessment. Direct observation is considered the gold standard for measuring hand hygiene compliance rather than self-reported measures [63]. There is a paucity of literature regarding the method of compliance measurement with other IPC practices. The other available literature regarding compliance measurement is for chlorhexidine baths, where direct observation may be similar to self-reported measures [64].

The limitations of our study include that it is a single-centre study, there was no separate control group, and a single observer measured the compliance. Nevertheless, our observation protocol enabled measurement of compliance of all the IPC practices and the compliance rates measured in the study are similar to those from other studies from multiple countries [28,33,50]. Every hospital or even different ICUs within a hospital may need protocols individualized to its needs, and our study provides a framework for developing such protocols.

## Conclusion

This study developed a set of VAP and CLABSI IPC nursing practices through a participatory approach. Implementing the multimedia tool, which encompasses the IPC practices, improved compliance with many practices.

## Ethics statement

The Institutional Ethical Committee (IEC) approved the study (INT/IEC/2021/SPL-1728).

## Funding sources

None.

## Conflict of interest statement

None declared.
