# Supplementary material for: A multimedia tool for infection prevention and control practices in the intensive care unit: a participatory interventional before–after study
Source: Infect Prev Pract. 2024 Dec 5;7(1):100423. doi: 10.1016/j.infpip.2024.100423 (PMC11728882; doi:10.1016/j.infpip.2024.100423)
Supplement: Multimedia component 1 [file mmc1.docx]

**Supplementary Table S1**

Delphi results for infection prevention and control practices for ventilator-associated pneumonia obtained from 12 experts on a 5-point Likert scale: 1, strongly disagree; 2, disagree; 3, neither agree nor disagree; 4, agree; 5, strongly agree.

CVI, content validity index, caluclated by dividing the number of responses recorded as 4 or 5 by the number of experts (12); Exp, experts.

| S. no. | Infection prevention and control practices | Exp 1 | Exp 2 | Exp 3 | Exp 4 | Exp 5 | Exp 6 | Exp 7 | Exp 8 | Exp 9 | Exp10 | Exp 11 | Exp 12 | CVI |
| --- | --- | --- | --- | --- | --- | --- | --- | --- | --- | --- | --- | --- | --- | --- |
| Hand hygiene | |  |  |  |  |  |  |  |  |  |  |  |  |  |
| 1 | A Sanitizer bottle is available near each patient’s bed | 5 | 5 | 5 | 5 | 5 | 5 | 5 | 5 | 5 | 5 | 5 | 5 | 1 |
| 2 | Hand hygiene is performed using an alcohol-based hand rub (if not visibly soiled) or using soap and water (if visibly soiled) | 5 | 5 | 5 | 5 | 5 | 5 | 5 | 5 | 5 | 5 | 5 | 5 | 1 |
| Oral care | |  |  |  |  |  |  |  |  |  |  |  |  |  |
| 3 | Patients requiring continued intubation > 12 hours are provided oral care 6 hourly | 5 | 5 | 5 | 5 | 5 | 4 | 5 | 5 | 4 | 5 | 4 | 4 | 1 |
| 4 | The endotracheal cuff pressure is assessed, followed by aspiration of oropharyngeal secretions before beginning the oral care procedure | 5 | 5 | 5 | 4 | 5 | 5 | 5 | 5 | 5 | 5 | 5 | 5 | 1 |
| 5 | Oral care is provided with one toothbrush and toothpaste OR an artery forceps and a gauze impregnated with chlorhexidine gluconate to cleanse the teeth, tongue, and mucosal surfaces | 5 | 5 | 3 | 3 | 4 | 3 | 1 | 4 | 5 | 5 | 5 | 4 | 0.67 |
| 6 | Thereafter oro-pharyngeal area is sucked with a suction set | 4 | 5 | 5 | 4 | 5 | 5 | 5 | 5 | 4 | 5 | 4 | 5 | 1 |
| Daily bath with Chlorhexidine Gluconate | |  |  |  |  |  |  |  |  |  |  |  |  |  |
| 7 | Patient (if appropriate post gestational age) admitted to the ICU is bathed/sponged with chlorhexidine solution | 5 | 5 | 5 | 4 | 5 | 5 | 5 | 5 | 4 | 5 | 5 | 4 | 1 |
| 8 | Bathing/sponging is performed daily from admission to discharge | 5 | 5 | 5 | 4 | 5 | 5 | 5 | 5 | 5 | 5 | 5 | 5 | 1 |
| 9 | The whole body surface except for the face, surgical wound and drain tube sites is cleaned in sequence | 5 | 5 | 5 | 4 | 4 | 4 | 2 | 5 | 4 | 5 | 4 | 4 | 0.92 |
| Assessment of ETT cuff pressure | |  |  |  |  |  |  |  |  |  |  |  |  |  |
| 10 | ETT cuff pressure is maintained >20 cmH_2_o & < 30 cmH_2_o with No/minimal leak | 5 | 5 | 5 | 5 | 5 | 5 | 3 | 5 | 4 | 5 | 4 | 4 | 0.92 |
| 11 | ETT cuff pressure is monitored in each shift in addition to following intubation, after manipulation or adjustment of the endotracheal tube, and when clinically indicated for an air leak or a loss of tidal volume | 5 | 5 | 5 | 5 | 4 | 5 | 5 | 5 | 4 | 4 | 4 | 4 | 1 |
| Hypertonic Saline Nebulization | |  |  |  |  |  |  |  |  |  |  |  |  |  |
| 12 | Nebulizing pediatric patients with 3% hypertonic saline | 5 | 5 | 5 | 5 | 2 | 5 | 5 | 5 | 4 | 5 | 4 | 4 | 0.92 |
| 13 | Hypertonic sodium chloride 3% solution is delivered to the ventilator by placing a T-piece connector into the inspiratory arm of the respiratory circuit around 20 cm from the endotracheal tube | 3 | 3 | 5 | 4 | 2 | 5 | 4 | 5 | 4 | 4 | 4 | 4 | 0.75 |
| Mechanical Ventilation and Care | |  |  |  |  |  |  |  |  |  |  |  |  |  |
| 14 | The ventilator-humidification device is routinely inspected | 5 | 5 | 5 | 5 | 4 | 5 | 5 | 5 | 5 | 5 | 5 | 5 | 1 |
| 15 | The proper temperature setting of the heated humidifier system is maintained | 5 | 5 | 5 | 5 | 4 | 5 | 5 | 5 | 4 | 5 | 5 | 5 | 1 |
| 16 | Condensation from the patient circuit is removed (when required) | 5 | 5 | 5 | 5 | 5 | 5 | 5 | 4 | 4 | 5 | 5 | 4 | 1 |
| 17 | The required water level in the heated humidifier is maintained | 5 | 5 | 5 | 4 | 4 | 5 | 5 | 5 | 5 | 5 | 4 | 5 | 1 |
| Patient Position in Bed | |  |  |  |  |  |  |  |  |  |  |  |  |  |
| 18 | Patients’ position (if not contraindicated) is changed two hourly | 5 | 5 | 4 | 3 | 5 | 5 | 5 | 5 | 4 | 5 | 4 | 4 | 0.92 |
| 19 | Pillows/positioning blocks are used to provide right/left lateral position | 5 | 5 | 5 | 3 | 4 | 5 | 5 | 5 | 4 | 5 | 4 | 5 | 0.92 |
| Chest Physiotherapy | |  |  |  |  |  |  |  |  |  |  |  |  |  |
| 20 | Before ET suctioning, manual application of a fine oscillatory movement combined with compression to the patient’s chest wall is provided | 5 | 3 | 5 | 4 | 4 | 4 | 3 | 5 | 4 | 5 | 4 | 4 | 0.83 |
| 21 | Two appropriately skilled staff perform the suction procedure | 5 | 4 | 5 | 5 | 5 | 5 | 4 | 4 | 3 | 5 | 5 | 4 | 0.92 |
| 22 | Hand hygiene performed before beginning the procedure | 5 | 5 | 5 | 4 | 5 | 5 | 5 | 5 | 5 | 5 | 5 | 5 | 1 |
| 23 | Use of personal protective equipment | 5 | 4 | 4 | 3 | 2 | 4 | 5 | 5 | 5 | 4 | 4 | 4 | 0.83 |
| 24 | Suction equipment and appropriate-sized suction catheter (2 x ETT internal diameter size) are assembled beforehand | 5 | 5 | 5 | 5 | 5 | 5 | 5 | 5 | 5 | 5 | 4 | 4 | 1 |
| 25 | Perform suction of oral secretions as completely as possible, and do not let the suction tube extend into the oropharyngeal space | 5 | 5 | 5 | 2 | 5 | 5 | 5 | 5 | 4 | 5 | 4 | 4 | 0.92 |
| 26 | Position the patient in a semi-Fowler's position (30° bed head elevation) if not contraindicated | 5 | 5 | 5 | 5 | 4 | 5 | 5 | 5 | 4 | 5 | 4 | 4 | 1 |
| 27 | Pre-oxygenate the patient for two minutes with 100% oxygen (if not contraindicated) | 3 | 5 | 5 | 3 | 4 | 5 | 5 | 5 | 4 | 5 | 5 | 4 | 0.83 |
| 28 | Sedate the patient with bolus (if agitated) | 5 | 5 | 3 | 3 | 4 | 5 | 5 | 4 | 4 | 4 | 5 | 4 | 0.83 |
| 29 | Use In-line suctioning in patients on ventilators for more than 12 hours | 4 | 5 | 3 | 5 | 2 | 5 | 4 | 5 | 5 | 5 | 5 | 4 | 0.83 |
| 30 | Use suction-support mode for suctioning | 3 | 5 | 3 | 5 | 5 | 3 | 5 | 5 | 5 | 5 | 4 | 5 | 0.75 |
| 31 | Use the clean hand/dirty hand technique to perform suction | 5 | 5 | 4 | 5 | 4 | 4 | 5 | 5 | 5 | 5 | 5 | 4 | 1 |
| 32 | Insert a suction catheter in the endotracheal tube until obstruction is felt | 2 | 5 | 5 | 2 | 4 | 4 | 5 | 5 | 5 | 5 | 5 | 4 | 0.83 |
| 33 | Suction applied only (100mmHg) during catheter withdrawal and for no longer than 5 seconds in each attempt | 5 | 5 | 5 | 4 | 4 | 5 | 5 | 5 | 4 | 5 | 5 | 4 | 1 |
| 34 | The suction catheter is kept sterile when used for repeated suctions during the same suction episode | 4 | 5 | 5 | 4 | 4 | 5 | 5 | 2 | 4 | 4 | 5 | 4 | 0.92 |
| 35 | Wrap and Discard the suction catheter if contaminated during the procedure or upon completion of the procedure | 5 | 5 | 5 | 5 | 5 | 5 | 5 | 5 | 5 | 5 | 5 | 5 | 1 |
| 36 | Manually ventilate the patient or apply vital capacity breath (if advised) for lung recruitment after suctioning | 4 | 5 | 3 | 5 | 5 | 4 | 5 | 4 | 4 | 5 | 3 | 4 | 0.83 |
| 37 | Limiting the time for suctioning <15 seconds | 5 | 5 | 3 | 5 | 4 | 4 | 3 | 5 | 5 | 5 | 5 | 4 | 0.83 |

**Supplementary Table S2**

Delphi results for infection prevention and control practices for central-line-associated bloodstream infections obtained from 14 experts on a 5-point Likert scale: 1, strongly disagree; 2, disagree; 3, neither agree nor disagree; 4, agree; 5, strongly agree.

CVI, content validity index, caluclated by dividing the number of responses recorded as 4 or 5 by the number of experts (14); Exp, experts.

| S. no | Infection prevention and control practices | Exp1 | Exp2 | Exp3 | Exp4 | Exp5 | Exp 6 | Exp7 | Exp8 | Exp 9 | Exp10 | Exp 11 | Exp12 | Ex 13 | Exp14 | Item CVI |
| --- | --- | --- | --- | --- | --- | --- | --- | --- | --- | --- | --- | --- | --- | --- | --- | --- |
| 1 | Hand hygiene and clean gloving before IV injections | 5 | 5 | 5 | 5 | 4 | 5 | 5 | 4 | 4 | 5 | 4 | 5 | 5 | 5 | 1 |
| 2 | Scrub the hub with a 70% alcohol swab before accessing it | 5 | 5 | 5 | 5 | 5 | 5 | 5 | 3 | 5 | 5 | 4 | 5 | 5 | 5 | 0.92 |
| 3 | Sterile stopper connected to ports which are not in use | 5 | 5 | 5 | 5 | 5 | 5 | 5 | 5 | 5 | 5 | 5 | 5 | 5 | 5 | 1 |
| 4 | Aspirate from lumen before drug administration | 5 | 5 | 5 | 4 | 4 | 5 | 5 | 5 | 4 | 5 | 5 | 5 | 5 | 5 | 1 |
| 5 | Any difficulty in flushing the line is checked | 5 | 5 | 5 | 5 | 4 | 5 | 5 | 4 | 5 | 5 | 5 | 5 | 5 | 5 | 1 |
| 6 | Intactness of the transparent dressings is checked | 4 | 5 | 5 | 5 | 5 | 4 | 5 | 4 | 5 | 5 | 4 | 5 | 5 | 5 | 1 |
| 7 | Daily assessment for central line removal | 4 | 5 | 5 | 5 | 5 | 4 | 5 | 4 | 5 | 4 | 4 | 5 | 5 | 5 | 1 |
| 8 | The CVC insertion site is assessed once every shift (for signs of infection) | 5 | 5 | 5 | 5 | 4 | 4 | 5 | 4 | 5 | 4 | 4 | 5 | 5 | 5 | 1 |

*Observation sample sheet*

*Part I. Patient notes/Nurse report*

| S. no. | Interventions | Yes | No |
| --- | --- | --- | --- |
| 1 | Patients requiring continued intubation >12 hours are provided oral care 6 hourly |  |  |
| 2 | Patient (if appropriate post gestational age) admitted to the ICU is bathed/sponged with chlorhexidine solution |  |  |
| 3 | Patient (if appropriate post gestational age) admitted to the ICU is bathed/sponged with chlorhexidine solution |  |  |
| 4 | The whole body surface except for the face, surgical wound and drain tube sites is cleaned in sequence |  |  |
| 5 | ETT cuff pressure is maintained >20 cmH_2_O & <30 cmH_2_O with no/minimal leak |  |  |
| 6 | ETT cuff pressure is monitored in each shift in addition to following intubation, after manipulation or adjustment of the endotracheal tube, and when clinically indicated for an air leak or a loss of tidal volume |  |  |
| 7 | Nebulizing pediatric patients with 3% hypertonic saline |  |  |
| 8 | The ventilator-humidification device is routinely inspected |  |  |
| 9 | Patients’ position (if not contraindicated) is changed two hourly |  |  |
| 10 | Daily assessment for central line removal |  |  |
| 11 | The central line insertion site is assessed once every shift (for signs of infection) |  |  |

*Part II. Bedside observation*

| S. no. | Interventions | Yes | No |
| --- | --- | --- | --- |
| 1 | A Sanitizer bottle is available near each patient’s bed |  |  |
| 2 | Hypertonic sodium chloride 3% solution is delivered to the ventilator by placing a T-piece connector into the inspiratory arm of the respiratory circuit around 20 cm from the endotracheal tube |  |  |
| 3 | The proper temperature setting of the heated humidifier system is maintained |  |  |
| 4 | Condensation from the patient circuit is removed (when required) |  |  |
| 5 | The required water level in the heated humidifier is maintained |  |  |
| 6 | Pillows/positioning blocks are used to provide right/left lateral position |  |  |
| 7 | Intactness of the transparent dressings is checked |  |  |

*Part III. Procedures*

| S. no. | Interventions | Yes | No |
| --- | --- | --- | --- |
| 1 | Hand hygiene is performed using an alcohol-based hand rub (if not visibly soiled) or using soap and water (if visibly soiled) |  |  |

*Oral care*

| S. no. | Interventions | Yes | No |
| --- | --- | --- | --- |
| 1 | The endotracheal cuff pressure is assessed, followed by aspiration of oropharyngeal secretions before beginning the oral care procedure |  |  |
| 2 | Oral care is provided with one toothbrush and toothpaste OR an artery forceps and a gauze impregnated with chlorhexidine gluconate to cleanse the teeth, tongue, and mucosal surfaces |  |  |
| 3 | Thereafter oro-pharyngeal area is sucked with a suction set |  |  |

*Endotracheal suctioning*

| S. no. | Interventions | Yes | No |
| --- | --- | --- | --- |
| 1 | Before ET suctioning, manual application of a fine oscillatory movement combined with compression to the patient’s chest wall is provided |  |  |
| 2 | Two appropriately skilled staff perform the suction procedure |  |  |
| 3 | Hand hygiene performed before beginning the procedure |  |  |
| 4 | Use of personal protective equipment |  |  |
| 5 | Suction equipment and appropriate-sized suction catheter (2 × ETT internal diameter size) are assembled beforehand |  |  |
| 6 | Perform suction of oral secretions as completely as possible, and do not let the suction tube extend into the oropharyngeal space |  |  |
| 7 | Position the patient in a semi-Fowler's position (30° bed head elevation) if not contraindicated |  |  |
| 8 | Pre-oxygenate the patient for two minutes with 100% oxygen (if not contraindicated) |  |  |
| 9 | Sedate the patient with bolus (if agitated) |  |  |
| 10 | Use In-line suctioning in patients on ventilators for more than 12 hours |  |  |
| 11 | Use suction-support mode for suctioning |  |  |
| 12 | Use the clean hand/dirty hand technique to perform suction |  |  |
| 13 | Insert a suction catheter in the endotracheal tube until obstruction is felt |  |  |
| 14 | Suction applied only (100 mmHg) during catheter withdrawal and for no longer than 5 seconds in each attempt |  |  |
| 15 | The suction catheter is kept sterile when used for repeated suctions during the same suction episode |  |  |
| 16 | Wrap and discard the suction catheter if contaminated during the procedure or upon completion of the procedure |  |  |
| 17 | Manually ventilate the patient or apply vital capacity breath (if advised) for lung recruitment after suctioning |  |  |
| 18 | Limiting the time for suctioning <15 seconds |  |  |

*Central line drug administration*

| S. no. | Interventions | Yes | No |
| --- | --- | --- | --- |
| 1 | Hand hygiene and clean gloving performed before IV injections |  |  |
| 2 | The central line lumen (three-way connector) is cleaned every time with 70% alcohol swab before accessing it for drug administration |  |  |
| 3 | A sterile stopper is connected to all ports, which are not in use |  |  |
| 4 | The lumen is aspirated before flushing the line |  |  |
| 5 | Any difficulty flushing the line or the line sluggishness or occlusion is notified |  |  |
